# An Inducible Cre-*lox* System to Analyze the Role of LLO in *Listeria monocytogenes* Pathogenesis

**DOI:** 10.3390/toxins12010038

**Published:** 2020-01-07

**Authors:** Brittney N. Nguyen, Daniel A. Portnoy

**Affiliations:** 1 Graduate Group in Microbiology, University of California, Berkeley, CA 94720, USA; brittneynguyen@berkeley.edu; 2 Department of Molecular and Cell Biology, University of California, Berkeley, CA 94720, USA; 3 Department of Plant and Microbial Biology, University of California, Berkeley, CA 94720, USA

**Keywords:** pathogenesis, cytotoxicity, pore-forming toxin, cholesterol-dependent cytolysin, vaccine

## Abstract

Listeriolysin O (LLO) is a pore-forming cytolysin that allows *Listeria monocytogenes* to escape from phagocytic vacuoles and enter the host cell cytosol. LLO is expressed continuously during infection, but it has been a challenge to evaluate the importance of LLO secreted in the host cell cytosol because deletion of the gene encoding LLO (*hly*) prevents localization of *L. monocytogenes* to the cytosol. Here, we describe a *L. monocytogenes* strain (*hly*^fl^) in which *hly* is flanked by *loxP* sites and Cre recombinase is under the transcriptional control of the *L. monocytogenes actA* promoter, which is highly induced in the host cell cytosol. In less than 2 h after infection of bone marrow-derived macrophages (BMMs), bacteria were 100% non-hemolytic. *hly*^fl^ grew intracellularly to levels 10-fold greater than wildtype *L. monocytogenes* and was less cytotoxic. In an intravenous mouse model, 90% of bacteria were non-hemolytic within three hours in the spleen and eight hours in the liver. The loss of LLO led to a 2-log virulence defect in the spleen and a 4-log virulence defect in the liver compared to WT *L. monocytogenes*. Thus, the production of LLO in the cytosol has significant impact on the pathogenicity of *L. monocytogenes*.

## 1. Introduction

The field of microbial pathogenesis and the study of virulence factors has been guided for decades by Molecular Koch’s Postulates, which stipulate that inactivation of a gene encoding a suspected virulence factor should lead to measurable loss of virulence, and replacement of the gene should restore pathogenicity [1]. Although targeted gene deletions are invaluable in determining the function of genes and pathways, there remain circumstances in which it is not possible to generate viable deletion mutants, or deletion of a gene encoding multiple functions precludes analysis of later functions. The latter is the case for the gene encoding Listeriolysin O (*hly*) of *Listeria monocytogenes*.

*L. monocytogenes* is a Gram-positive facultative intracellular pathogen that specifically replicates in the cytosol of host cells. In order to reach the host cell cytosol, *L. monocytogenes* must first escape from the phagocytic entry vacuole, which requires the secreted pore-forming cytolysin Listeriolysin O (LLO) [2,3]. In the cytosol, *L. monocytogenes* replicates and produces an actin nucleation factor (ActA) to move intracellularly and form protrusions that are engulfed by neighboring cells and resolved into double-membraned vacuoles. Again, LLO is required for escape from these secondary vacuoles [4,5]. 

LLO belongs to a large family of cholesterol-dependent cytolysins (CDCs) which also includes: Perfringolysin of *Clostridium perfringens,* Streptolysin O of *Streptococcus pyogenes,* and Pneumolysin of *Streptococcus pneumoniae* [6]. Importantly, LLO is the only CDC produced by an intracellular pathogen and its roles in pathogenesis are distinct and unique compared to related CDCs [7].

Though the only clearly established role of LLO during infection is inside of *L. monocytogenes-*containing vacuoles, LLO is continuously secreted inside host cells. The continuous secretion of LLO has the potential to form pores in the host cell membrane and cause cell death. Mechanisms have been identified that suppress LLO activity within the host cytosol, lessening its potential toxicity, including: reduced activity at the neutral pH of the cytosol compared to the acidic pH of the vacuole, degradation by the proteasome, and translational repression by the 5’ coding sequence [8,9,10,11]. Additionally, LLO co-opts host endocytosis machinery for removal of LLO from the plasma membrane [12]. Mutations that abolish any of these control mechanisms increase *L. monocytogenes* cytotoxicity [13]. However, other than cell death, these studies do not address the potential activities that LLO has when secreted in the cytosol.

Despite the numerous mechanisms that suppress LLO activity in the cytosol, it is still not clear whether LLO is suppressed to the point where it has no activity. Unfortunately, whether LLO secreted in the cytosol has any effects has been difficult to address because of the early requirement for LLO, although strategies have been developed to examine this question. To show that LLO was required for escape from primary single-membrane and secondary double-membrane vacuoles, Gedde et al. noncovalently coupled purified LLO to Δ*hly L. monocytogenes* and observed that the bacteria were able to escape from the primary vacuole, but because of their inability to produce more LLO they became trapped in secondary vacuoles [4]. Similarly, Dancz et al. demonstrated that *L. monocytogenes* expressing IPTG-inducible LLO remain trapped in vacuoles until addition of IPTG [5]. Czuczman et al. studied *L. monocytogenes* in HeLa cells, in which LLO is not required for escape from the vacuole, and concluded that LLO causes localized plasma membrane damage that allows *L. monocytogenes* to hijack the cell efferocytosis machinery for cell-to-cell spread [14]. Others have observed that LLO disrupts SUMOylation, modifies histones, and causes mitochondrial fragmentation during infection by simply adding purified LLO exogenously to cells [15,16,17,18,19]. These studies have all tried to circumvent the requirement for LLO in the vacuole, but the diversity in the techniques used and their limitations makes it a challenge to integrate the conclusions into a complete picture. Additionally, these studies have not addressed the effects of these activities in mice.

We have developed a tool for studying LLO that does not circumvent the requirement for LLO in vacuolar escape. We engineered a strain of *L. monocytogenes* that initially produces LLO, allowing it to escape from the vacuole. After escape from the vacuole *hly* is excised by Cre-*lox-*mediated DNA recombination and the strain becomes a Δ*hly* mutant. Here, we report rapid excision of *hly* in bone marrow-derived macrophages (BMMs) and in a mouse model. In BMMs, LLO secreted in the cytosol contributes to significant amounts of cytotoxicity. *hly*^fl^ grew to a level 10-fold higher than wildtype (WT) *L. monocytogenes* in BMMs, likely due to a reduction in cell death. 

## 2. Results

### 2.1. Use of hly^fl^ in Cultured Cells

#### 2.1.1. Cre-*lox* Allows for Rapid Excision of hly during Infection of Macrophages

**Figure 1:**
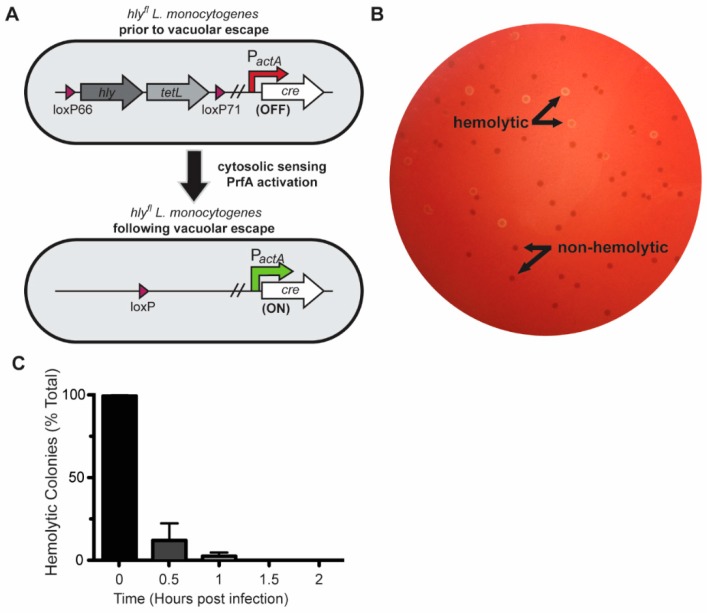
*hly* is excised in the *hly*^fl^ strain in bone marrow-derived macrophages (BMMs) through DNA recombination. (**A**) Schematic of *hly*^fl^ *L. monocytogenes* strain. The *hly* and *tetL* (tetracycline resistance) genes are flanked by *loxP* sites. Cre recombinase is controlled by the *actA* promoter. Recombination between *loxP* sites leads to the excision of the DNA encoding *hly* and *tetL* (1); (1): Reprinted from Cell Host & Microbe, Vol 23/Issue 6, Chen Chen, Brittney N. Nguyen, Gabriel Mitchell, Shally R. Margolis, Darren Ma, Daniel A. Portnoy, The Listeriolysin O PEST-like Sequence Co-opts AP-2-Mediated Endocytosis to Prevent Plasma Membrane Damage during Listeria Infection, 786–795., Copyright (2018), with permission from Elsevier. (**B**) Hemolytic and non-hemolytic colonies of *hly*^fl^. (**C**) To quantify the excision of *hly*, BMMs were infected with *hly*^fl^ and both hemolytic and non-hemolytic colonies were enumerated by plating bacteria on blood-agar media at different timepoints. Mean and SD of data pooled from three independent experiments are shown.

To study the role of LLO secreted in the cytosol during infection, we engineered a strain of *L. monocytogenes*, called *hly*^fl^, to excise the gene encoding LLO, *hly*, following escape of *L. monocytogenes* from the phagocytic vacuole. Specifically, *loxP* sites were inserted into the *L. monocytogenes* chromosome to flank *hly* and an adjacent gene, *tetL*, which provides tetracycline resistance. Cre recombinase, which mediates DNA recombination between *loxP* sites, was inserted into the chromosome using the pPL2 integrative vector, and expressed under the control of the *L. monocytogenes actA* promoter, which is relatively inactive prior to vacuolar escape of *L. monocytogenes* and becomes highly expressed in the cytosol (Figure 1A). Thus, this strain is able to produce LLO initially to facilitate escape from the phagocytic vacuole but, once in the cytosol, *hly* is excised and LLO production ceases. To determine the efficiency of the system, BMMs were infected with *hly*^fl^ *L. monocytogenes* and bacteria from the infected cells were recovered at different time points and plated on blood-agar media. Secreted LLO causes rapid β-hemolysis and *L. monocytogenes* colonies that secrete LLO can be easily identified (Figure 1B). Prior to infection, *hly*^fl^ *L. monocytogenes* were grown in broth containing tetracycline to select against low-level excision of *hly* and *tetL*. By 30 min post-infection almost 90% of recovered colony-forming units (CFU) were non-hemolytic (Figure 1C). By 60 min post-infection, 98% of recovered CFU were non-hemolytic and by 90 min post-infection all colonies were non-hemolytic. Therefore, the excision of *hly* is rapid and complete during infection of BMMs.

#### 2.1.2. LLO Secreted in the Cytosol Affects Intracellular Growth and Contributes to Cytotoxicity

**Figure 2:**
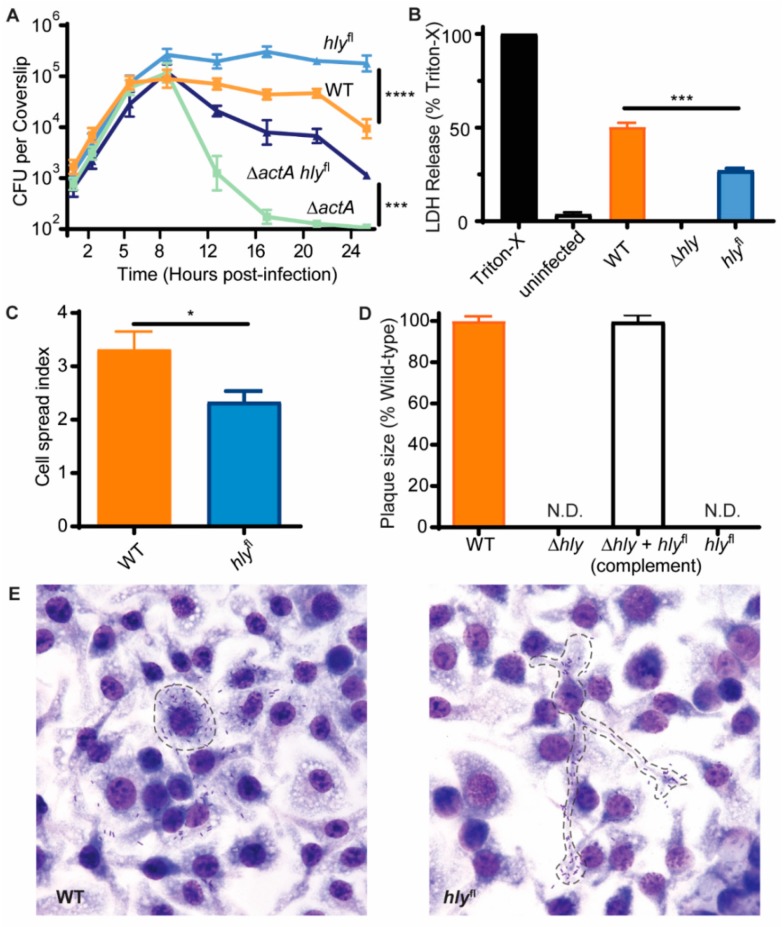
*hly*^fl^ grew intracellularly and had reduced cytotoxicity but had a defect in cell-to-cell spread. (**A**) BMMs were infected at an MOI of 0.17 with *L. monocytogenes* and intracellular CFU were enumerated at different times. Three biological replicates were measured in each experiment, and experimental results were combined. For Δ*actA* and Δ*actA hly*^fl^ *n* = 6. For WT and *hly*^fl^ *n* = 9. Mean and SEM are shown. Results were log transformed and means were compared using Holm–Sidak’s multiple comparisons test. (**B**) BMMs were infected with *L. monocytogenes* at an MOI of 4 for 24 h. Lactate dehydrogenase (LDH) release was measured and values were normalized according to lysis with 1% Triton-X 100 representing 100% LDH release and the condition with the smallest mean value representing 0% LDH release. The average of three technical replicates per experiment, from three independent experiments, are presented. Means were compared using an unpaired *t*-test. (**C**) BMMs were infected with *L. monocytogenes* and cell-to-cell spread was quantified 5 h post-infection. WT *n* = 67, *hly*^fl^ *n* = 60. Means were compared using an unpaired *t*-test. (**D**) L2 cells were infected with *L. monocytogenes* at an MOI of 0.1. Plaque size was measured after 3 days and values were normalized to WT plaque size. Mean and SEM for data pooled from three independent experiments is shown. WT *n* = 19, Δ*hly + hly*^fl^ *n* = 14. N.D. indicates no plaques were detectable. (**E**) Representative images of the BMMs infected with *L. monocytogenes* for cell-to-cell spread analysis in (**C**). Dashed lines are used to indicate the initially-infected cells.

To determine whether secretion of LLO by *L. monocytogenes* in the cytosol affects the growth of the bacteria in cells, intracellular growth was evaluated in BMMs. During the first five hours of infection, *hly*^fl^ *L. monocytogenes* grew identically to WT *L. monocytogenes* and Δ*actA*, which is defective in actin-based motility and therefore defective in cell-to-cell spread (Figure 2A). However, after five hours of infection, the growth of the strains diverged. Between five and twenty-four hours of infection, the number of WT *L. monocytogenes* plateaued and then declined, but the number of recovered *hly*^fl^ *L. monocytogenes* increased to 10-fold more than the maximum of WT and remained elevated, suggesting that secretion of LLO in the cytosol negatively impacts growth of WT.

WT *L. monocytogenes* has the ability to spread to and replicate in neighboring cells. If *hly*^fl^ has a defect in escape from secondary vacuoles that would limit its ability to replicate in neighboring cells, the difference in growth between WT and *hly*^fl^ may reflect both the effects of LLO secreted in the cytosol and growth following cell-to-cell spread. To analyze the effects of LLO in the cytosol without complication by cell-to-cell spread, we compared growth of *hly*^fl^ in a Δ*actA* background (Δ*actA hly*^fl^) to Δ*actA*, which is defective in actin-based motility and therefore defective in cell-to-cell spread. During the first eight hours of infection, Δ*actA* and Δ*actA hly*^fl^ grew similarly to WT. However, between eight and twenty-four hours, the number of Δ*actA* bacteria decreased dramatically, whereas Δ*actA hly*^fl^ decreased much less—having as much as 100-fold more bacteria than Δ*actA*. The rapid loss of Δ*actA* CFU, which is due to the influx of gentamicin [20], was partially rescued by deletion of *hly* in the cytosol, indicating that the decline of Δ*actA* in cells is partly LLO dependent.

The ability of LLO to form pores in cholesterol-containing cell membranes is well documented. We hypothesized that the growth of WT *L. monocytogenes* could be restricted by LLO-induced cytotoxicity because LLO has the potential to bind to the cell membrane and cause cell death. To quantify the amount of cell death caused by *L. monocytogenes* infection, cytotoxicity was measured by lactate dehydrogenase (LDH) release assay (Figure 2B). After a 24 h infection of BMMs, 43% of cells were killed by WT *L. monocytogenes* infection. Only 22% of cells were killed by *hly*^fl^ *L. monocytogenes* infection, indicating that LLO secreted in the cytosol contributes significantly to cytotoxicity during infection.

Previously, a requirement for LLO in escaping double-membraned phagocytic vacuoles after cell-to-cell spread was demonstrated using IPTG-inducible LLO [5]. Because *hly*^fl^ should delete *hly* in the cytosol of the first cell it infects, we expected it to also be defective in escape from secondary vacuoles. To examine the fate of *hly*^fl^, we performed cell spreading and plaque assays to measure cell-to-cell spread (Figure 2C–E). The number of cells in infectious foci were quantified for BMMs infected with WT and *hly*^fl^ for 5 h. On average, WT *L. monocytogenes* spread to two to three neighboring cells over a 5 h period, while *hly*^fl^ was only found in an average of two neighboring cells (Figure 2C). Furthermore, WT *L. monocytogenes* was often well distributed in cells of an infectious focus, indicating that bacterial replication had occurred in neighboring cells following cell-to-cell spread. In contrast, infectious foci of *hly*^fl^ were often observed with one macrophage harboring the majority of bacteria and limited numbers of bacteria in the surrounding cells, suggesting that *hly*^fl^ spread to neighboring cells, but had a subsequent vacuolar escape defect (Figure 2E). To assess the impact on cell-to-cell spread resulting from *hly* deletion over a longer time period, L2 cells were infected with WT and *hly*^fl^ *L. monocytogenes.* WT *L. monocytogenes* forms plaques in a monolayer of L2 cells by spreading from cell-to-cell. Both *hly*^fl^ and Δ*hly L. monocytogenes* were unable to form plaques, indicating that they cannot efficiently spread cell-to-cell. Δ*hly* that was complemented with *hly*^fl^ but missing Cre recombinase, and therefore unable to excise *hly*, was restored to WT *L. monocytogenes* levels of plaque formation (Figure 2D). These results suggest that though LLO is not required for growth in cells, its continued production contributes to cell-to-cell spread.

### 2.2. Use of hly^fl^ in Mice

#### 2.2.1. *hly* is Excised In Vivo and Its Excision Reduces Virulence

**Figure 3:**
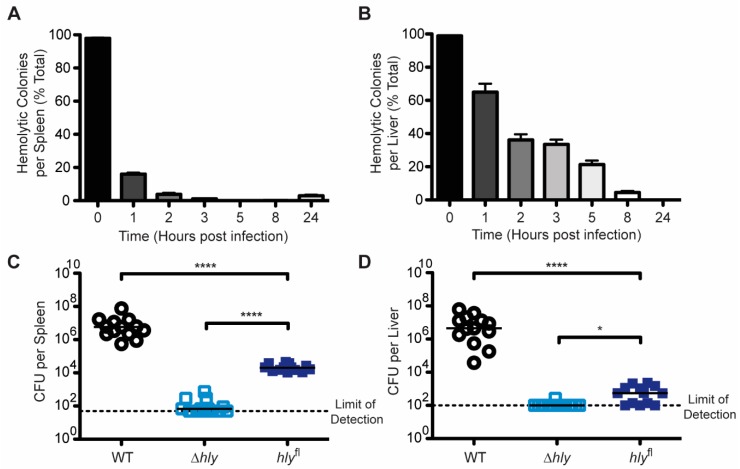
*hly*^fl^ recombines in vivo and is attenuated in mice. (**A**,**B**) C57BL/6J mice were infected with 1 × 10^5^ CFU of *hly*^fl^ *L. monocytogenes.* For each timepoint, hemolytic and non-hemolytic colonies recovered from the (**A**) spleen and (**B**) liver were enumerated. % Total hemolytic colonies was calculated for each mouse. Mean and SEM are shown. Data are pooled from two independent experiments. For each timepoint, *n* = 6. (**C**,**D**) CD-1 mice were infected with 1 × 10^5^ CFU of *L. monocytogenes.* Then, 48 h post-infection, CFU from the (**C**) spleen and (**D**) liver were enumerated. Data are pooled from three independent experiments and medians are shown. *n* = 13. Means were compared using Holm–Sidak’s multiple comparisons test.

To quantify the efficiency of the *hly*^fl^ Cre-*lox* system in vivo, C57BL/6J mice were infected intravenously with 10^5^ CFU of *hly*^fl^ *L. monocytogenes* (Figure 3). Hemolytic capacity of the inoculum was verified by plating on blood agar. At 1, 2, 3, 5, 8, and 24 h post-infection, bacteria were recovered from the spleen and liver and plated on blood agar and both hemolytic and non-hemolytic CFU were enumerated. In the spleen (Figure 3A), less than 20% of bacteria were hemolytic one hour post-infection. Hemolytic bacteria represented less than 2% of the population three hours post-infection and were nearly undetectable five hours post-infection. Then, 24 h post-infection, a small population of hemolytic bacteria were detected in the spleen (see discussion). In the liver (Figure 3B), excision of *hly* was slower than in the spleen. One hour post-infection, only 35% of bacteria were non-hemolytic; eight hours post-infection 95% of bacteria were non-hemolytic and hemolytic colonies were undetectable by 24 h post-infection. 

To determine the importance of LLO after escape of *L. monocytogenes* from the initial vacuole, we characterized *hly*^fl^ *L. monocytogenes* using a mouse model of virulence. CD-1 mice were infected intravenously with 10^5^ CFU of *L. monocytogenes* and 48 h post-infection, CFU from the spleen and liver were enumerated (Figure 3). WT *L. monocytogenes* grew to 10^7^ CFU in both the spleen and liver, while Δ*hly L. monocytogenes* was extremely attenuated with bacteria from the spleen and liver near or below the limit of detection in most mice. Interestingly, *hly*^fl^ *L. monocytogenes* had a moderate level of attenuation. 10^4^ CFU were recovered from the spleen, representing a statistically-significant 3-log reduction in virulence compared to WT, although a smaller reduction in virulence than that of Δ*hly*. In the liver, less than 10^3^ CFU were recovered.

#### 2.2.2. Vaccination with *hly*^fl^ Confers Protective Immunity

**Figure 4:**
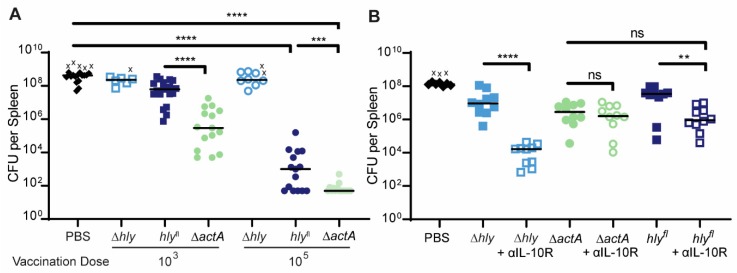
Vaccination with *hly*^fl^ confers protective immunity in mice. (**A**) C57BL/6J mice were vaccinated intravenously with 10^3^ or 10^5^ CFU of *L. monocytogenes.* Then, 4 weeks post-vaccination, mice were challenged intravenously with 5 × 10^4^–1 × 10^5^ CFU of WT *L. monocytogenes.* Three days post-challenge, CFU were enumerated from the spleen. Data are pooled from two to three independent experiments. Means were compared using Holm–Sidak’s multiple comparisons test. (**B**) C57BL/6J mice were vaccinated intravenously with 10^3^ Δ*actA* or *hly*^fl^ *L. monocytogenes* or 10^8^ Δ*hly L. monocytogenes* +/− treatment with αIL-10R antibody. Mice were challenged intravenously with 5 × 10^4^ CFU of WT *L. monocytogenes.* Three days post-challenge, CFU were enumerated from the spleen. Data are pooled from two independent experiments. Means were compared using Holm–Sidak’s multiple comparisons test. X indicates a mouse that died from the WT challenge before organs could be harvested.

The two requirements for a vaccine are safety and efficacy. In Figure 3, we showed that *hly*^fl^ is highly attenuated, and thus satisfies the safety requirement. To test the efficacy of *hly*^fl^ *L. monocytogenes* as a vaccine, a protection study was performed. Protection of *hly*^fl^ was compared to protection conferred by Δ*actA*, which is well established as an attenuated and effective vaccine strain [21], and Δ*hly*, which does not confer strong protection. C57BL/6J mice were vaccinated with either 10^3^ or 10^5^*L. monocytogenes*. Four weeks post-vaccination, the mice were challenged with a lethal dose of WT *L. monocytogenes*. Three days post-challenge, CFU from the spleen were enumerated (Figure 4A). Vaccination with 10^5^ CFU of *hly*^fl^ provided 5-logs of protection, albeit less protection than Δ*actA*, while vaccination with 10^5^ CFU of Δ*hly* did not confer protection. However, vaccination with 10^3^ *hly*^fl^ was not as protective as 10^3^ Δ*actA*. 

As vaccination with vacuole-confined Δ*hly* is inhibited by the secretion of the immunosuppressive cytokine IL-10 [22], we hypothesized that *hly*^fl^ is also inhibited by IL-10 because it is defective in cell-to-cell spread and remains confined in secondary vacuoles. To test this hypothesis, we performed protection studies using an IL-10 receptor blocking antibody (αIL-10R), which improves the protective capacity of Δ*hly* [22]. Indeed, administration of αIL-10R improved the protective capacity of Δ*hly* and *hly*^fl^ but not Δ*actA L. monocytogenes* (Figure 4B). Furthermore, the protection conferred by *hly*^fl^ was improved to levels similar to those conferred by vaccination with Δ*actA.* Thus, *hly*^fl^ is a highly attenuated strain of *L. monocytogenes* capable of inducing protective immunity, though its protection is reduced compared to Δ*actA* likely due to IL-10 induction. 

## 3. Discussion

As a member of the cholesterol-dependent cytolysin (CDC) family of pore-forming toxins, LLO is unique as the only CDC that is secreted by an intracellular pathogen and therefore the only CDC that primarily acts on cells from within. Therefore, LLO likely affects cells in ways that extracellular CDCs do not. Yet, because deletion of *hly* prevents study of the effects of LLO in the cytosol, other studies have utilized the application of exogenous purified LLO to study its effects [15,16,17,18,19]. Here, we described a *L. monocytogenes* strain that uses a Cre-*lox* system to delete *hly* following vacuolar escape. This strain, *hly*^fl^, became 100% non-hemolytic less than 1.5 h after infection of macrophages (Figure 1C) and replicated to high numbers in the cytosol (Figure 2A,E). *hly* excision after vacuolar escape allows the study of LLO functions in the cytosol separate from its role in vacuolar escape.

### 3.1. Insights into the Effects of LLO during Infection

LLO is a pore-forming toxin that oligomerizes and forms pores in cholesterol-containing membranes, including the host cell vacuolar and plasma membranes [23,24]. Secretion of LLO in the cytosol has the potential to damage the host cell plasma membrane. Although multiple mechanisms limit LLO damage to the cell plasma membrane, it has been difficult to establish whether these mechanisms are entirely effective in preventing cell death [7]. Here, we showed that *L. monocytogenes* that does not produce LLO in the cytosol replicates to greater numbers in macrophages and is less cytotoxic. The revelation that LLO secreted in the cytosol is cytotoxic makes it curious that *L. monocytogenes* continuously secretes LLO. It is possible that the continuous production of LLO is necessary to ensure a rapid escape from secondary vacuoles, as defects that reduce cell-to-cell spread are highly attenuated. 

It is also possible that the innate immune response to LLO-induced cell death contributes to pathogenesis. In the host cell cytosol, infrequent lysis of *L. monocytogenes* induces pyroptotic cell death via the AIM2 inflammasome [25]. Some forms of cell death are thought to inhibit the generation of protective immunity. Strains of *L. monocytogenes* engineered to induce pyroptosis, necrosis or apoptosis inhibit the generation of protective immunity [26]. Thus, it is likely that LLO-induced cell death also affects the immune response to *L. monocytogenes* infection. The type of cell death induced by LLO and its effects on the innate and adaptive immune responses to *L. monocytogenes* merit further study.

Analysis of the kinetics of *hly* deletion, specifically the more rapid deletion in the spleen compared to the liver, revealed differences in the environments experienced by *L. monocytogenes*. CD169+ macrophages that are localized to the marginal zone of the spleen and dendritic cells are thought to be the first splenic cell types infected by *L. monocytogenes* upon intravenous inoculation [27,28]. Three hours post-infection, the majority of *L. monocytogenes* in the spleen are trapped within CD169+ macrophages [27]. Following infection with *hly*^fl^, bacteria in the spleen became non-hemolytic at a rate similar to in BMMs, with almost complete loss of hemolytic capacity three hours post-infection, suggesting that infection of BMMs closely models infection in the spleen with respect to activation of virulence genes. However, bacteria became non-hemolytic at a much slower rate in the liver, with greater than 20% of bacteria remaining hemolytic at 5 h post-infection and complete loss of hemolysis between eight and 24 h post-infection. In the liver, four hours after infection with *L. monocytogenes* 100% of infected cells are tissue-resident macrophages, known as Kupffer cells [29]. Bacteria that are not killed by the Kupffer cells can transfer to hepatocytes, which become heavily infected [30]. Over the next couple days, infected Kupffer cells die by necroptosis; infiltrating neutrophils and monocyte-derived macrophages lyse infected hepatocytes and become the primarily infected cells [30,31,32]. It is possible that *hly*^fl^ became non-hemolytic slowly in the liver because *actA* expression was not efficiently upregulated in Kupffer cells, and Cre-*lox* recombination only occurred after the bacteria were transferred to hepatocytes, neutrophils and/or monocyte-derived macrophages. If this is the case, it would be interesting to understand why the intracellular environment of a Kupffer cell does not activate *actA* expression like other cells. Alternatively, the bacteria that became non-hemolytic during the first 8 h of infection could represent the population of bacteria inside Kupffer cells, hepatocytes and/or infiltrating neutrophils and monocyte-derived macrophages, and the bacteria that remained hemolytic may represent an extracellular population of bacteria in the liver. A small population of extracellular bacteria associated with nonparenchymal cells in the liver six hours after infection has been previously identified, though it is not clear whether these bacteria became extracellular following lysis of infected hepatocytes, or whether they never infected cells [33].

### 3.2. Limitations of hly^fl^

Although we have successfully employed *hly*^fl^ to demonstrate the contribution of LLO to cytotoxicity in macrophages, this system is limited by the fact that loss of LLO is permanent and not conditional to the environment of the cytosol. As a result, *hly*^fl^ became trapped in secondary vacuoles and was defective in cell-to-cell spread (Figure 2C–E). Bacteria that are released from the cytosol upon cell lysis also behave like LLO-minus mutants. The inability to escape subsequent vacuoles likely explains the attenuation of *hly*^fl^ in mice (Figure 3C,D), and why *hly*^fl^ vaccination was improved by αIL-10R antibody (Figure 4B). 

An additional complication of *hly*^fl^ is that Cre-*lox* recombination is susceptible to inactivation. A previous study in which a transposon library was generated in a strain of *L. monocytogenes* with Cre-*lox* identified transposon insertions in the *actA* promoter driving *cre* expression and *loxP* sites that prevented recombination. We observed a population of hemolytic bacteria in the spleen that expanded between eight- and 24 h post-infection. We isolated several colonies of hemolytic bacteria 24 h post-infection and reinfected BMMs, and bacteria were 100% hemolytic five hours post-infection (data not shown). It is possible that these hemolytic bacteria are the progeny of a founding bacterium that had a mutation in its Cre-*lox* machinery that prevented recombination from occurring.

In the future, the ideal tool to study the cytosolic effects of LLO in mice would not secrete LLO in the host cell cytosol but could secrete LLO upon entry into secondary cells to continue the life cycle. Nevertheless, we believe *hly*^fl^ is well suited for studying LLO secreted in the cytosol in cells, and insights gained from study of *hly*^fl^ in cells can be translated to the whole animal setting using various mouse models.

## 4. Conclusions

Cre-*lox* recombination has been a popular tool for the study of plants and mice for many decades [34,35,36]. In bacteria, its use has been more limited, and it has only been used a few times in *L. monocytogenes*. Previously in *L. monocytogenes*, Cre-*lox* was used to generate a strain that cannot replicate following activation of the *actA* promoter by flanking essential genes near the origin of replication with *loxP* sites and driving Cre expression with the *actA* promoter [37]. This strain is highly attenuated and potently activates the CD8+ T-cell response and thus is a candidate vaccine-delivery system [38]. In another instance, a strain of *L. monocytogenes* that deletes *actA* in the host cell cytosol was used to show that ActA expressed in the host cell cytosol contributes to cell-to-cell spread and simultaneously allows *L. monocytogenes* to avoid xenophagy [39]. This works represents the first use of Cre-*lox* recombination to study the function of a virulence factor that is active at temporally and spatially distinct periods. We believe that this system has the promise to uncover many effects of LLO secreted in the cytosol and could also uniquely contribute to better understanding the cellular responses to membrane damage from an intracellularly secreted pore-forming toxin. 

## 5. Materials and Methods

### 5.1. Construction of hly^fl^

*hly*^fl^ was constructed by integrating two plasmids, one encoding *hly* and *tetL* flanked by *loxP* sites and the other encoding cre downstream of the *actA* promoter, into a Δ*hly* strain of *L. monocytogenes.* The *hly* and *tetL* genes were cloned into pPL1 and flanked by *lox66/lox71 loxP* sites such that Cre expression resulted in excision of the region flanked by *loxP* sites (fl). The resulting plasmid (pPL1-*hly*^fl^) was transformed into SM10 *E. coli*. The cre recombinase gene was previously engineered downstream of the *actA* promoter in pPL2e, yielding the plasmid pPL2e-*actA-cre*, which was also transformed into SM10 E. Coli. Transconjugation was performed to integrate both plasmids into Δ*hly L. monocytogenes* in a stepwise manner. First, pPL1-*hly*^fl^ was transconjugated with Δ*hly L. monocytogenes* and transconjugate colonies that were resistant to streptomycin (200 μg/mL) and chloramphenicol (7.5 μg/mL) were selected. Second, Δ*hly* pPL1-hlyfl was transconjugated with pPL2e-*actA-cre*, and transconjugate colonies resistant to streptomycin, chloramphenicol, and erythroymycin (1 μg/mL) were selected. Similarly, Δ*actA hly*^fl^ was engineered by transconjugating pPL1-*hly*^fl^ and pPL2e-actA-cre into Δ*actA*Δ*hly*. The *hly*^fl^ complement strain was engineered by transconjugating Δ*hly* with pPL1-*hly*^fl^. 

A control pPL1 plasmid (pPL1-*tetL*^fl^) encoding *tetL*, but not *hly*, flanked by *loxP* sites was engineered by excising *hly* from pPL1-*hly*^fl^. The Δ*hly* control strain was engineered by transconjugating Δ*hly* with pPL1-*tetL*. The WT control strain was engineered by transconjugating WT with pPL1-*tetL*^fl^. The Δ*actA* control strain was engineered by transconjugating Δ*actA* with pPL1-*tetL*^fl^.

### 5.2. Bacterial Culture

Strains used in this study are listed in Table S1. Bacteria were grown overnight at 37 °C in Brain-Heart Infusion (BHI; BD, Sparks, MD, USA) containing 200 μg/mL streptomycin (GoldBio, St. Louis, MO, USA), and bacteria with *tetL* were additionally grown in 2 μg/mL tetracycline (GoldBio, St. Louis, MO, USA). Overnight cultures were diluted 1:200 and grown in BHI containing streptomycin (for bacteria without *tetL*) or streptomycin and tetracycline (for bacteria with *tetL*) at 37 °C, shaking, to an optical density of 0.5. These cultures were then pelleted by centrifugation and resuspended in phosphate-buffered solution (PBS; Gibco, Paisley, UK) containing 9% glycerol. These cultures were then aliquoted and frozen at −80 °C. Aliquots were thawed and used directly for experiments. 

### 5.3. Preparation of M-CSF

The 3T3 cell media was prepared using Dulbecco’s Modified Eagle Media (DMEM, Gibco, Grand Island, NY, USA) with 10% FBS (Seradigm, US Origin), 1% L-Glutamine (Corning, Manassas, VA, USA), and 1% Sodium pyruvate (Corning, Manassas, VA, USA), with or without 1× Penicillin Streptomycin Solution (“Pen/Strep”; Corning, Manassas, VA, USA). The 10^7^ M-CSF-producing 3T3 cells were seeded into a T75 flask with 20mL media containing Pen/Strep and grown at 37 °C 5% CO_2_ (Day 1). To split cells, media was aspirated, cells were washed with warm PBS, and incubated with 0.05% Trypsin-EDTA (Gibco, Grand Island, NY, USA) for five minutes at 37 °C. On day 4, cells were split to a T225 in 50 mL media containing Pen/Strep. On day 7, cells were split to five T225 flasks in media without Pen/Strep. On day 9 or 10, when cells when cells were 100% confluent, the five T225 flasks were split into 25 T225 flasks and grown until 100% confluent (about 3 days) and an additional two days (about 5 days total) in media without Pen/Strep. On day 14 or 15, supernatant was removed from all flasks, filter sterilized with a 0.2 µM bottle filter, and stored at 4 °C. Then, 50 mL fresh media without Pen/Strep was added back to each T225 and flasks were incubated an additional 3 days. On day 17 or 18, supernatants were collected as before, and combined with the previous supernatants. Supernatants were stored at −20 °C and used as the source of M-CSF for bone marrow-derived macrophage preparation and culture.

### 5.4. Bone Marrow-Derived Macrophage Culture

BMM growth media was prepared using high glucose DMEM (Thermo Fisher Scientific) with 20% Fetal Bovine Serum (Seradigm), 1% L-glutamine (Corning), 1% Sodium pyruvate (Corning), 14mM 2-Mercaptoethanol (Gibco; Grand Island, NY, USA), and 10% 3T3 cell supernatant (from M-CSF-producing 3T3 cells). Macrophages were prepared from the femurs of C57BL/6J mice. Femurs were isolated, sterilized with 70% ethanol, and crushed with a mortar and pestle in BMM growth media. Cells were strained through a 70µM filter and distributed into ten 150-mm non-TC dishes in 30mL BMM culture medium. An additional 30mL BMM culture medium was added at day 3. After cells were incubated for a total of seven days at 37 °C with 5% CO_2_, cells were harvested and frozen at −80 °C in BMM culture medium with 10% Fetal Bovine Serum (Seradigm) and 10% DMSO (Sigma, St. Louis, MO, USA) added.

### 5.5. Blood Agar Media

Blood agar media was prepared using the following recipe for 1L of media: 5 g yeast extract, 10 g tryptone (BTS, Richmond, CA, USA), 10 g NaCl (Fisher, Fair Lawn, NJ, USA), 10.48 g MOPS (Sigma, St. Louis, MO, USA), 1 g activated charcoal, 5 g SuperPure Agar (BTS Richmond, CA, USA), 957.5 mL MilliQ water (Millipore Sigma, USA), and 10 N NaOH to bring pH to 7.3. Media was autoclaved and cooled prior to addition of 12.5 mL 304 g/L glucose-1-phosphate (Sigma, St. Louis, MO, USA) and 25 mL defibrinated Sheep’s blood (Hemostat Laboratories, Dixon, CA, USA).

### 5.6. Hemolysis in BMMs

A 60 mm non-TC dish with 15 12 mm glass coverslips was seeded with 3 × 10^6^ BMMs. The following day, the cells were infected with 2 × 10^5^ CFU *hly*^fl^ for 30 min. Then, cells were washed with PBS and BMM media with 50 µg/mL gentamicin was added. At each timepoint, coverslips were removed from the dish and placed in water to lyse the cells. Bacteria were plated on blood-agar media. Plates were incubated overnight at 37 °C and then transferred to 4 °C until halos surrounding hemolytic colonies were clear. Hemolytic and ahemolytic colonies were enumerated.

### 5.7. Hemolysis in Mice

C57BL/6J (The Jackson Laboratory, Bar Harbor, ME, USA) mice were infected with 1 × 10^5^ CFU of *hly*^fl^ *L. monocytogenes.* At 0, 1, 2, 3, 5, 8, and 24 h post-infection mice were euthanized (3 mice per timepoint) and spleens and livers were harvested, homogenized, and plated on blood-agar media. Plates were incubated overnight at 37 °C and then transferred to 4 °C until halos surrounding hemolytic colonies were clear. Hemolytic and ahemolytic colonies were enumerated.

### 5.8. Lactate Dehydrogenase (LDH) Assay

BMMs were seeded into a 24-well plate with 5 × 10^5^ BMMs/well. The following day, cells were infected with 2 × 10^6^ CFU L. monocytogenes for 30 min. Then, cells were washed with PBS and BMM media containing 5% FBS and 50μg/mL gentamicin was added to wells. Then, 24 h post-infection LDH assay was performed as previously described [25].

### 5.9. Intracellular Growth Curves

In total, 3 × 10^6^ BMMs were plated in 60 mm non-TC-treated Petri dishes with 15 12 mm glass coverslips in each dish. The following day, two dishes per strain were infected with 5 × 10^5^ CFU (MOI = 0.17) and intracellular growth curves were performed as described previously [40].

### 5.10. Plaque Assay

Six-well plates were seeded with 1.2 × 10^6^ L2 cells per well. The plaque assay was performed as previously described [41].

### 5.11. Cell Spreading Assay

BMMs were seeded into a 24-well plate with glass coverslips in each well, at 3.5 × 10^5^ cells/well. Three coverslips were infected for each strain. Then, 30 min post-infection, cells were washed with PBS and BMM media containing 50 μg/mL gentamicin (Sigma-Aldrich, St. Louis, MO, USA) was added. At five hours post-infection, coverslips were removed, and cells were fixed with 100% methanol and stained using Diff-Quik stain. Coverslips were mounted onto glass slides using Permount (Fisher Chemical). Light microscopy was performed using a BZ-X700 microscope (KEYENCE, Osaka, Japan) and a 60× objective lens. Cell spread index was calculated by counting the number of cells in an infectious focus containing 5 or more bacteria and subtracting one cell so that the cell spread index represents the number of cells that were spread to from the initially infected cell. Analysis was performed blindly. In total, 60 *hly*^fl^ and 67 WT infectious foci were analyzed.

### 5.12. Animal Use Ethics Statement

All animal work was done in strict accordance with university regulations. Protocols were reviewed and approved by the Animal Care and Use Committee at the University of California, Berkeley AUP-2016-05-8811. Date of approval: 11 February 2016.

### 5.13. Virulence in Mice

Eight-week-old female CD-1 mice (Charles River Laboratories, Wilmington, MA, USA) were infected intravenously with 1 × 10^5^ CFU in 200 µL PBS. Forty-eight hours post-infection, the mice were euthanized, and spleens and livers were harvested, homogenized in 0.1% IGEPAL CA-630 (Sigma, St. Louis, MO, USA) in water, and plated on LB agar (Fisher, Fair Lawn, NJ, USA) with 200 µg/mL streptomycin for enumeration of bacterial burdens. 

### 5.14. Vaccination of Mice 

Eight-to-ten-week-old female C57BL/6J mice (The Jackson Laboratory) were vaccinated by intravenous injection of *L. monocytogenes* in 200 µL PBS. Four weeks post-vaccination, mice were challenged with 5 × 10^4^ CFU WT *L. monocytogenes* injected intravenously in 200 µL PBS. Three days post-challenge, mice were euthanized, and spleens and livers were harvested, homogenized in 0.1% IGEPAL CA-630 (Sigma), and plated on LB Agar with 200 µg/mL streptomycin for enumeration of bacterial burdens. Mice treated with αIL-10R antibody (Clone 1B1.3A, Bio X Cell, West Lebanon, NH, USA) were injected with 250 µg of antibody in 100 µL PBS two hours prior to vaccination. 

### 5.15. Statistical Analysis

Data were analyzed using GraphPad Prism 8 (GraphPad, San Diego, CA, USA, 2019). For mouse CFU experiments, data were log transformed prior to performing statistical analysis. * indicates *p* < 0.05; ** indicates *p* < 0.01; *** indicates *p* < 0.001; **** indicates *p* < 0.0001; ns indicates no statistical significance. 

## Supplementary Materials

The following are available online at https://www.mdpi.com/2072-6651/12/1/38/s1, Table S1: Strains.
