# Supplementary material for: An Inducible Cre-lox System to Analyze the Role of LLO in Listeria monocytogenes Pathogenesis
Source: Toxins (Basel). 2020 Jan 7;12(1):38. doi: 10.3390/toxins12010038 (PMC7020405; doi:10.3390/toxins12010038)
Supplement: Supplementary file 1 [file toxins-12-00038-s001.pdf]

Supplementary Materials: An Inducible Cre-*lox* System to Analyze the Role of LLO in *Listeria monocytogenes* Pathogenesis

Brittney N. Nguyen and Daniel A. Portnoy

Table 1. Strains.

Strain Name	Strain Background	Plasmid 1	Plasmid 2	Strain Number	Reference
hly[fl]	Δ*hly*	pPL1-*hly*[fl]	pPL2e-*actA-cre*	DP-L6648	[12]
Δ*hly* + *hly*[fl] (complement)	Δ*hly*	pPL1-*hly*[fl]	-	DP-L6647	[12]
WT	WT 10403S (DP-L6234; DP-L4056 phage-cured)	pPL1-*tetL*[fl]	-	DP-L6896	This study
Δ*hly*	Δ*hly* (DP-L6236; DP-L4027 phage-cured)	pPL1-*tetL*[fl]	-	DP-L6897	This study
Δ*actA*	Δ*actA* (DP-L6235; DP-L4029 phage-cured)	pPL1-*tetL*[fl]	-	DP-L6894	This study
Δ*actA hly*[fl]	Δ*actA*Δ*hly* phage-cured (DP-L6237)	pPL1-*hly*[fl]	pPL2e-*actA-cre*	DP-L6883	This study
pPL1-*hly*[fl]	SM10			DP-E6869	This study
pPL1-*tetL*[fl]	SM10			DP-E6892	This study
pPL2e-*actA-cre*	SM10			DP-E6233	[36]
